# Chromosome-level genome assembly of *Cheilinus chlorourus* (Bloch, 1791) (Perciformes: Labridae)

**DOI:** 10.1038/s41597-025-05288-y

**Published:** 2025-07-02

**Authors:** Jiangyong Qu, Xueying Yang, Zhuoran Song, Shuang Wang, Zhikai Xing, Lijun Wang, Xumin Wang

**Affiliations:** School of Life Science, Yantai University, Yantai, Shandong 264005 China

**Keywords:** Molecular biology, Computational biology and bioinformatics

## Abstract

In the classification of marine fish, the Labridae family ranks second in terms of species diversity and plays a vital role in coral reef ecosystems, comprising over 600 species across 82 genera. Despite its significance for ecological and evolutionary studies, genomic research on this group has lagged, resulting in a shortage of data, particularly regarding high-quality chromosome-level genome assemblies. To address this gap, this study focused on *Cheilinus chlorourus* from the Labridae family and successfully achieved a chromosome-level genome assembly. By integrating Illumina, PacBio, and Hi-C sequencing data, we assembled a genome measuring 940.36 Mb, with 926.86 Mb (98.56%) of the gene assembly organized into 21 chromosomes. A total of 29,213 protein-coding genes (PCGs) were identified, and 79.93% of these genes were functionally annotated. With this high-quality genome assembly, future investigations into the functional genomics and ecology of *C. chlorourus* will have a solid scientific foundation.

## Background & Summary

The Labridae family, renowned for its exceptional species diversity in marine ecosystems, ranks second in species richness among marine fish. It comprises over 600 species across 82 genera^1^. The Labridae family is notable for its significant morphological and ecological diversity, showcasing an impressive array of colors, shapes, and sizes^2,3^. This diversity makes it an excellent candidate for research on adaptive radiation, ecological niche differentiation, and the processes of sexual selection among species^4^. Additionally, fish from the Labridae family play a crucial role in coral reef ecosystems by maintaining biodiversity and ecological balance within these environments^5^.

*Cheilinus chlorourus* (Bloch, 1791) is a member of the genus *Cheilinus* in the order Perciformes and the family Labridae. It is widely distributed in coral reef ecosystems^6^. It is widely distributed across the Indian Ocean and the Pacific Ocean, as well as in the waters surrounding Taiwan and Hainan Island in China^7,8^. This species primarily inhabits coral reef areas characterized by mixed reef sand or regions with abundant seagrass^1,3^. The feeding habits of *C. chlorourus* are diverse, with a diet that mainly consists of fish, mollusks, crustaceans, polychaetes, and sea urchins^1^. *C. chlorourus* is a vital species in coral reef ecosystems^9^, playing an essential role in preserving biodiversity, material cycling, and ecological stability^10^. It is also a significant indicator for evaluating the evaluating the ecological health of coral reef. However, research on the biological traits of *C. chlorourus* is limited, particularly in the field of genomics. Like many other coral reef fish, there is a severe lack of genomic resources for *C. chlorourus*, with no genomic data currently available. Furthermore, genomic research on coral fishes, in general, has progressed slowly, resulting in insufficient data, especially regarding high-quality chromosome-scale genome sequences. This lack of information hinders a comprehensive understanding of evolutionary processes, adaptive radiation, and functional genomics in coral reef fish.

To address this research gap, this study integrated PacBio long-read sequencing, Illumina short-read sequencing, and Hi-C technology to assemble the first high-quality, chromosome-level genome of *C. chlorourus*. The resulting assembled genome measures 940.36 Mb, of which 926.86 Mb (98.56%) were anchored to 21 chromosomes. We identified 29,213 protein-coding genes (PCGs), 79.93% of which were functionally annotated. Furthermore, we discovered 6,661 non-coding RNAs (ncRNAs) and 468.4 Mb of repetitive elements. The genomic data from this research not only fill the gaps in the genomic information of *C. chlorourus* and provide a crucial foundation for understanding its biological characteristics, but also provide high-quality reference genomes for comparative studies of coral reef fish.

## Methods

### Sampling and sequencing

Samples were collected from the nearshore waters of Sanya, Hainan, China. Muscle tissue was isolated from the captured fish and used for DNA extraction, followed by genome sequencing and assembly. Immediately after collection, the muscle samples were rapidly frozen and preserved in liquid nitrogen to maintain their integrity until DNA extraction. The use of experimental animals was reviewed and approved by the Animal Welfare and Ethics Committee of Yantai University.

**Fig. 1:**
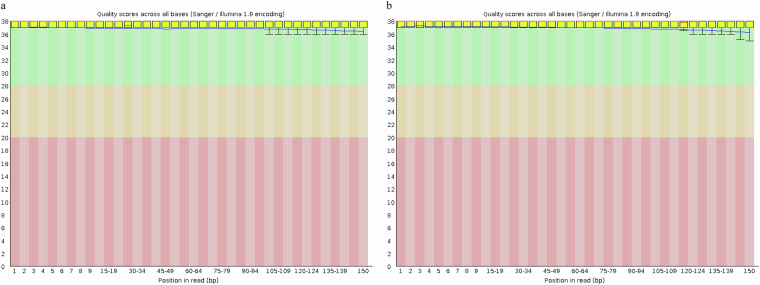
Base quality distribution of Illumina raw data. (**a**) R1, (**b**) R2. The horizontal axis represents the coordinates of the read bases, while the vertical axis indicates the base masses of the reads (Solexa Scale: 40 = Highest, −15 = Lowest). The box plot illustrates the distribution of mass values for all bases at each position. The red line indicates the median base mass, while the blue line represents the concatenation of the mean values at each position.

Muscle tissue DNA was isolated according to the manufacturer’s instructions using the E.Z.N.A^®^ Tissue DNA Kit (OMEGA, USA). The DNA was then fragmented into short segments ranging from 300 to 500 base pairs (bp) with a Covaris M220 device. The TruSeq^TM^ Nano DNA Sample Prep Kit (Illumina, USA) was employed to create a sequencing library containing 450 bp insert fragments. The libraries were quantified using the TBS-380 Picogreen technology from Invitrogen. Subsequently, high-throughput sequencing of the sample DNA was conducted on the Illumina NovaSeq 6000 platform^11^, generating 150 bp paired-end reads for genome surveys and base-level corrections. Following Illumina sequencing, approximately 22.05 Gb of initial sequence data were obtained (Table 1). Using FastQC software^12^, we analyzed the base-level quality of the Illumina raw sequencing data. The results indicated that all data had base quality scores within the green region, signifying high quality (Fig. 1). To enhance the accuracy of subsequent genome assembly, we opted to use Trimmomatic v0.39^13^ (https://www.usadellab.org/cms/index.php?page=trimmomatic) for quality control and trimming of the raw data. This step effectively removed adapter contamination and poor-quality read segments, ensuring robust downstream analyses. As a result, approximately 21.95 Gb of clean data was obtained.

**Table 1:** Statistics of second-generation sequencing data (Illumina sequencing and Hi-C sequencing).

Libraries types	Inter size (bp)	Raw data (Mb)	Clean data (Mb)	Q20 (%)	Q30 (%)
Illumina reads	450	22,046.6	21,951.8	99.02	97.13
Hi-C reads	450	128,962	118,091	98.76	96.86

After preparing the SMRTbell library, high-fidelity (HiFi) sequencing was performed on the PacBio Sequel II platform^14^. The raw sequencing data underwent several processing steps, including adapter removal, filtering of low-quality reads, and correction of sequencing errors. This process resulted in 35.34 Gb of data with a mean read length of 20.85 Kb (Table 2). DNA from an individual *C. chlorourus* was utilized to create a Hi-C library for chromosome-scale genome assembly^15,16^. Cells were exposed to formaldehyde to facilitate the cross-linking of DNA and proteins, thereby maintaining the stability of the DNA structure. The MboI restriction enzyme was employed to cleave the cross-linked DNA into fragments with sticky ends following cell lysis. The resulting DNA fragments underwent end repair and were simultaneously labeled with biotinylated oligonucleotides. Adjacent DNA fragments were then circularized through ligation using T4 DNA ligase. The cross-linked DNA-protein complexes were subsequently digested with protease to release the DNA. After purification, the DNA was fragmented to a size range of 500–700 bp. Streptavidin-coated magnetic beads were utilized to isolate biotin-labeled DNA, facilitating the construction of sequencing libraries compatible with next-generation sequencing platforms. The quantification and sequencing of the Hi-C libraries were conducted on the Illumina NovaSeq 6000 platform, generating data to support chromosome-scale genome assembly^17^. Following sequencing, the raw data underwent rigorous quality control, which included the removal of splice sequences, elimination of low-quality bases, and de-weighting. Approximately 118.09 Gb of high-quality clean data were obtained through this process (Table 1).

**Table 2:** PacBio HiFi sequencing data statistics table.

Reads Number	Reads Bases(bp)	Largest Length(bp)	N50 Length(bp)	N90 Length(bp)	Average Length(bp)
1,694,834	35,336,828,526	60,859	21,320	15,191	20,850

### Genome complexity assessment of *C. chlorourus*

**Fig. 2:**
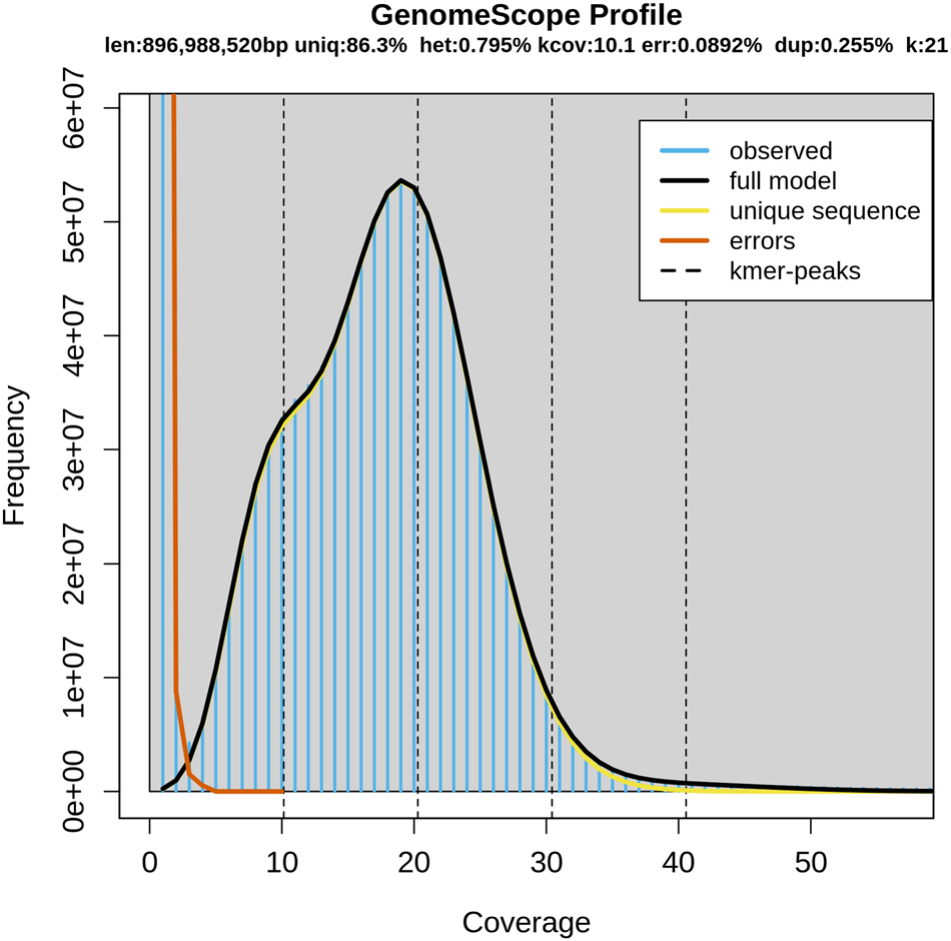
The genomic K-mer frequency distribution plot. (len: estimated genome size; uniq: proportion of non-repetitive sequences; het: heterozygosity; kcov: depth of heterozygous peak coverage; err: proportion of kmer generated by sequencing errors; dup: proportion of repetitive sequences; k: size of kmer used for evaluation).

Prior to genome assembly, K-mer statistical analysis was employed to estimate the genome size of *C. chlorourus*^18,19^. By analyzing the sequencing data using 21-mers, the estimated genome size was approximately 897.0 Mb, with the samples exhibiting a heterozygosity rate of 0.795% and a duplication rate of 0.255%. The substantial amount of non-repetitive sequences (unique) and the minimal error rate (error) indicated that the quality characteristics of the sequencing data were high (Fig. 2).

### Genome de novo assembly of *C. chlorourus*

Assemble three generations of PacBio HiFi long reads using the Hifiasm software^20^ (https://github.com/chhylp123/hifiasm). Hifiasm is a rapid and efficient de novo assembler specifically optimized for PacBio HiFi read lengths^20^. It processes all high-fidelity (HiFi) reads in memory for comparison and error correction. During this process, if a base in a read differs from the others and is supported by a minimum of three reads, it is identified as a single nucleotide polymorphism (SNP) and preserved; if this condition is not met, it is corrected as a sequencing error. This correction process eliminates most errors while preserving heterozygous variant information, significantly enhancing the accuracy of haplotype assembly. Based on the sequencing data, we have preliminarily constructed a genomic framework with a total length of approximately 940.36 Mb, which includes 39 scaffolds and a scaffold N50 of 42.47 Mb (Table 3). These results are consistent with the genome complexity estimates obtained from K-mer analysis.

**Table 3:** Comparison of the assembled genomes of *C. chlorourus* with those of *C. undulatus* and *L. mixtus*.

Features	Cheilinus undulatus^24^	Cheilinus chlorourus	Labrus mixtus^25^
Estimated genome size (Mb)	1,173.2	870.0	740.6
Contig number	329	103	894
Contig N50 (bp)	16,477,222	39,597,849	2,678,957
Scaffold number	45	39	324
Total length (bp)	1,170,280,289	940,359,651	740,579,663
Scaffold N50 (bp)	51,466,935	42,465,204	30,403,003
GC contents (%)	39.5	40.36	41
Number of chromosomes	24	21	24
Length of scaffolds anchored on chromosomes (Mb)	1,170.0 (99.98%)	926,86 (98.56%)	679.85 (94.23%)

### Chromosome-level genome assembly

**Fig. 3:**
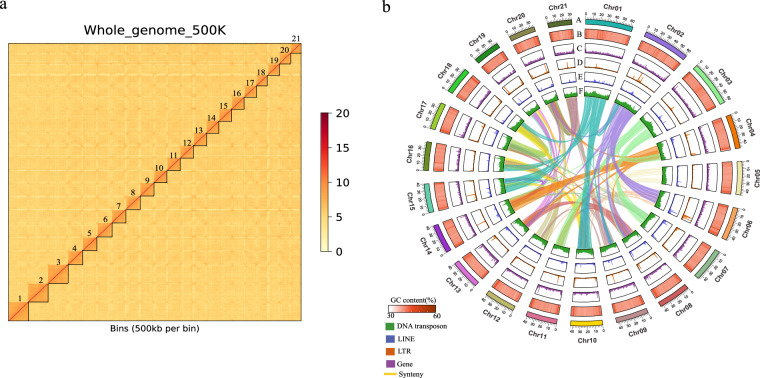
Hi - C assembly chromosome interaction heat map (**a**) and genome circle map of *C. chlorourus* (**b**). The circle map from the outside in: “A”represents chromosomes; “B”represents the GC content; “C”represents the gene density; “D”represents the LTR density; “E”represents the LINE density; “F”represents represents the DNA transposon density. Window size = 100 kb. Each central line in the circle represents connections between homologous gene pairs.

Hi-C technology-assisted scaffold construction was utilized in the genome assembly process^21^. The overlapping clusters were analyzed using ALLHIC (v0.9.8) software (https://github.com/tanghaibao/allhic) to assess their associations^22,23^. The principle of Hi-C assisted assembly is based on the idea that cis interactions are stronger than trans interactions, and that cis interactions are enhanced as the linear distance between elements decreases. This principle facilitates the clustering, sorting, and orientation of contigs or scaffolds, which is essential for assembling a chromosome-level genome. The assembly was conducted using 118.09 Gb of clean data obtained from the Hi-C library (Table 1). The resulting sequences, totaling 926.86 Mb (98.56%), were successfully anchored and assigned to 21 chromosomes, with sizes ranging from 30.07 Mb to 63.24 Mb (Tables 3, 4). Compared to its congeneric species member *Cheilinus undulatus* (GCA_018320785.1)^24^, the genome of *C. chlorourus* is smaller in size. This difference reflects variations in the evolutionary history of the genomes between species rather than disparities in sequencing quality. In terms of assembly quality, the *C. chlorourus* genome demonstrates superior continuity, with a contig N50 of 39.6 Mb, which surpasses that of *C. undulatus*. Additionally, the fewer scaffolds observed in this study indicate a more compact assembly structure. When compared to *Labrus mixtus* (GCA_963584025.1)^25^, a member of the same family, *C. chlorourus* exhibits significant advantages across all assembly metrics, further confirming the higher completeness and practical utility of its genomic sequence (Table 3). Furthermore, to evaluate the quality of the chromosome-level genome assembly, we generated a whole-genome Hi-C heat map (Fig. 3a). This heatmap clearly delineated all 21 chromosomes, with significantly stronger interaction signals along the diagonal compared to other regions, indicating a high-quality genome assembly.

**Table 4:** Assembly sequence length statistics.

ID	Length (bp)	GC_content	ID	Length (bp)	GC_content
Chr01	63,236,608	40.27%	Chr12	41,610,170	40.07%
Chr02	62,688,117	40.23%	Chr13	40,719,286	40.27%
Chr03	62,431,378	40.43%	Chr14	40,249,603	40.32%
Chr04	47,434,788	40.10%	Chr15	40,117,939	40.11%
Chr05	46,156,843	39.99%	Chr16	39,750,637	40.26%
Chr06	46,013,100	40.53%	Chr17	37,895,311	40.36%
Chr07	45,882,783	40.23%	Chr18	37,250,221	40.68%
Chr08	43,880,989	40.44%	Chr19	36,248,224	40.72%
Chr09	43,841,752	40.09%	Chr20	35,206,806	40.76%
Chr10	42,465,204	40.11%	Chr21	32,067,316	40.76%
Chr11	41,716,596	40.39%	—	—	—
Total (bp)	926,863,671	—

### Assessment of the genome assemblies

**Fig. 4:**
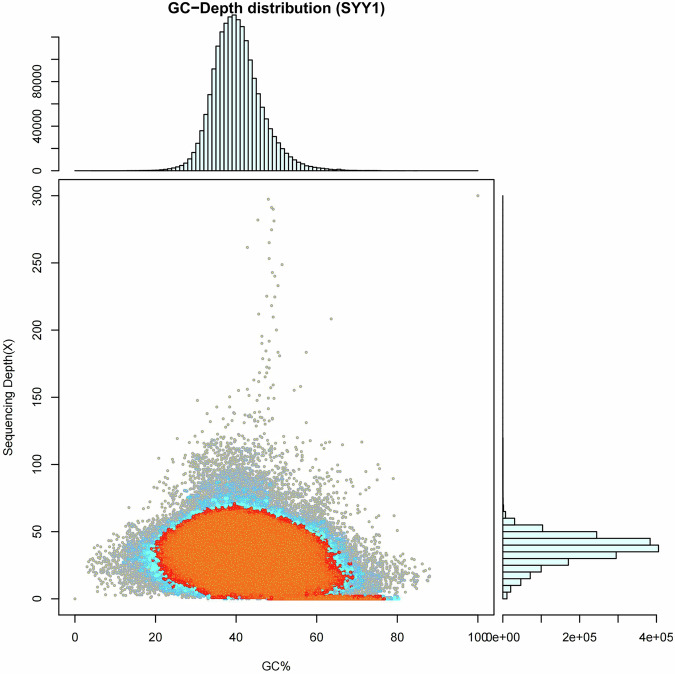
Graphical representation of the correlation between GC content and depth of sequencing. (The horizontal coordinates represent GC content, while the vertical coordinates indicate sequencing depth. The right side displays the distribution of sequencing depth, and the top shows the distribution of GC content. In this scatter plot, the red area highlights regions where the density of dots is relatively high).

To assess the quality of the genome assemblies, GC-Depth analysis was employed to identify potential biases in GC composition or non-target DNA contamination^26^. The sequencing reads were initially aligned to the constructed genomic sequences, followed by an analysis of both GC content and sequencing depth. Subsequently, correlation analyses between GC characteristics and sequencing depth were conducted. The findings indicated no significant GC bias, suggesting that the sequencing was performed with exceptional quality (Fig. 4). Furthermore, the integrity of the assembly was evaluated using Benchmarking Universal Single-Copy Orthologs (BUSCO v5.3.2) software^27,28^ (https://busco.ezlab.org/). The analysis revealed that out of 3,640 single-copy genes, 98.9% were found to be complete. Specifically, single-copy genes account for 97.9%, while duplicated genes make up 1.0%. The proportion of fragmented genes is 0.2%, and 0.9% of genes are missing in the assembled genome (Table 5). The low rates of duplications and deletions, combined with the high alignment rate of the raw sequencing data, collectively indicate that the quality of our assembly is high. The Merqury^29^ method was employed to evaluate the accuracy and completeness of the assembled genome. The quality value (QV) of the genome was determined to be 65.5965, with an error rate of 2.75642 e - 07. These assessment results further validated the completeness of the *C. chlorourus* genome assembly.

**Table 5:** Busco assessment results.

Type	Number	Percentage (%)
Complete BUSCOs (C)	3,602	98.9%
Complete and single-copy BUSCOs (S)	3,565	97.9%
Complete and duplicated BUSCOs (D)	37	1.0%
Fragmented BUSCOs (F)	9	0.2%
Missing BUSCOs (M)	29	0.9%
Total BUSCO groups searched	3,640	—

### Repetitive sequence analysis

Prior to identifying and functionally annotating coding genes within the genome, we first classified duplicated sequences in the *C. chlorourus* genome using both homology-based alignment and de novo prediction methods. Scattered repeats were identified using RepeatMasker software^30^. Tandem Repeats Finder (TRF)^31^ was employed to detect tandem repeat sequences in the DNA. RepeatMasker identifies scattered repeats by aligning sequences with a database of known repetitive elements, such as Repbase. TRF analyzes tandem repeat sequences by validating percentages and examining the frequency of insertions and deletions (InDels) in neighboring patterns, applying statistical criteria for identification. Ultimately, we identified 468.4 Mb of repetitive sequences, which included 325.3 Mb of scattered repeats and 143.1 Mb of tandem repeats, accounting for 49.8% of the entire assembled genome. Among the scattered repeats, DNA transposons (DNAs) constituted the largest portion, totaling 136.9 Mb, while short interspersed nuclear elements (SINEs) represented the smallest portion at 2.6 Mb, which is 0.28% of the entire genome (Table 6).

**Table 6:** Repeat elements in *C. chlorourus* genome.

Type	Number	Total Length (bp)	In Genome (%)
Interspersed repeats			
LTR	74,554	23,052,968	2.4515
DNA	740,847	136,940,465	14.5626
LINE	148,773	35,518,670	3.7771
SINE	20,917	2,607,877	0.2773
RC	36,818	8,690,801	0.9242
scRNA	0	0	0
Unknown	695,815	128,977,271	13.7157
Subtotal	1,717,724	325,308,952	34.5941
Tandem repeats			
TRF	289,345	80,186,546	8.5272
Minisatellite DNA	187,786	55,822,679	5.9363
Microsatellite DNA	53,820	7,083,298	0.7533
Subtotal	530,951	143,092,523	15.2168
Total	2,248,675	468,401,475	49.8109

### Prediction of non-coding genes

The identification of tRNA regions and their corresponding secondary structures was performed using tRNAscan-SE v2.0.7^32^, while rRNA sequence predictions were made through RNAmmer software^33^. A total of 2,765 tRNAs were identified with tRNAscan-SE, and 2,679 rRNA genes were predicted by RNAmmer. The prediction of miRNA, snRNA, and sRNA followed a similar workflow, beginning with comparative annotations against the Rfam database^34^ using Rfam software^35,36^. This was followed by the CMsearch program^37^, which employed default parameters to identify the final sRNAs, snRNAs, and miRNAs. In total, 619 snRNAs and 598 miRNAs were predicted (Table 7).

**Table 7:** Non-coding RNA result statistics.

	Type	Number	Average length (bp)	Total length (bp)	In Genome (%)
	tRNA	2,765	74	207,251	0.022
rRNA_de	5S	2,535	115	290,548	—
	5.8S	0	0	0	—
	18S	72	1,845	132,846	0.0992
	28S	72	7,076	509,493	—
rRNA_ho	5S	—	—	—	—
	5.8S	—	—	—	—
	18S	—	—	—	—
	28S	—	—	—	—
	sRNA	0	0	0	0
	snRNA	619	142	88,240	0.0094
	miRNA	598	75	45,064	0.0048

### Prediction of protein-coding genes

To perform gene prediction on the *C. chlorourus genome*, we integrated multiple approaches, including de novo prediction, comparison of homologous protein sequences, and analysis of transcriptome data. The reference genome of *C. undulatus* (GCA_018320785.1)^24^ was utilized to train the de novo gene prediction software AUGUSTUS v3.2.3^38^ (https://bioinf.uni-greifswald.de/augustus/). The predictions based on homology were conducted using protein sequences from *C. undulatus*, which were aligned with the *C. chlorourus* genome sequences via TBLASTn^39^. Poorly matched results were filtered out, and redundancies were removed. GeneWise v2.4.1^40^ (https://www.ebi.ac.uk/seqdb/confluence/display/THD/GeneWise) was then employed for precise comparisons to identify the coding and intron regions of the genes. Transcriptome data from muscle tissues were aligned with the genome sequences using TopHat v2.1.1^41^ (https://ccb.jhu.edu/software/tophat/index.shtml), and transcripts were assembled using Trinity v2.11.0^42^ (https://github.com/trinityrnaseq/trinityrnaseq/releases). The resulting gene sets were integrated using EVidenceModeler v1.1.1^43^ (https://evidencemodeler.github.io/). In total, 29,213 protein-coding genes were identified (Table 8). To validate the accuracy of gene prediction, we compared the green-tailed lipped fish genome with published genomes of eight other fish species: *C. undulatus*^24^, *L. mixtus*^25^, *Labrus bergylta*^44^, *Sebastes umbrosu*s^45^, *Pseudochaenichthys georgianus*^46^, *Notolabrus celidotus*^47^, *Acanthochromis polyacanthus*^48^, and *Stegastes partitus*^49^. The results demonstrated that key genomic features—including the number of protein-coding genes, average gene length, CDS length, and exon length—were comparable to those of other species, supporting the high quality of the genome assembly and annotation (Table 8).

**Table 8:** Statistical table of coding gene information.

Species	Gene number	Average gene length (bp)	Average CDS length (bp)	Average exon length (bp)	Average intron length (bp)	Average exons per gene
***C. chlorourus***	29,213	14,879.45	1,538.60	184.85	1,831.70	8.4
*C. undulatus*	23,316	30,378.47	1,829.37	293.37	2,676.84	10.5
*L. mixtus*	22,740	20,702.07	2,176.52	267.75	1,847.78	13.2
*L. bergylta*	23,510	19,105.06	1,838.40	313.67	1,527.99	10.5
*S. umbrosus*	23,881	22,843.00	1,839.73	279.00	1,882.33	10.5
*P. georgianus*	23,287	21,975.50	2,003.35	251.05	2,085.57	12.0
*N. celidotus*	22,740	23,263.18	1,995.87	269.30	2,010.07	11.9
*A. polyacanthus*	23,921	26,022.88	1,807.07	324.65	2,140.13	10.4
*S. partitus*	22,589	19,484.58	1,770.82	259.78	1,665.61	11.9

For the assembled genome sequences of the sequenced samples, the genomic features of *C. chlorourus* were visualized alongside the coding genes and the results of repeat predictions. Circular genome maps were generated using Circos software^50^, illustrating gene counts, repeat densities, and GC content (Fig. 3b).

### Functional annotation of genes

**Fig. 5:**
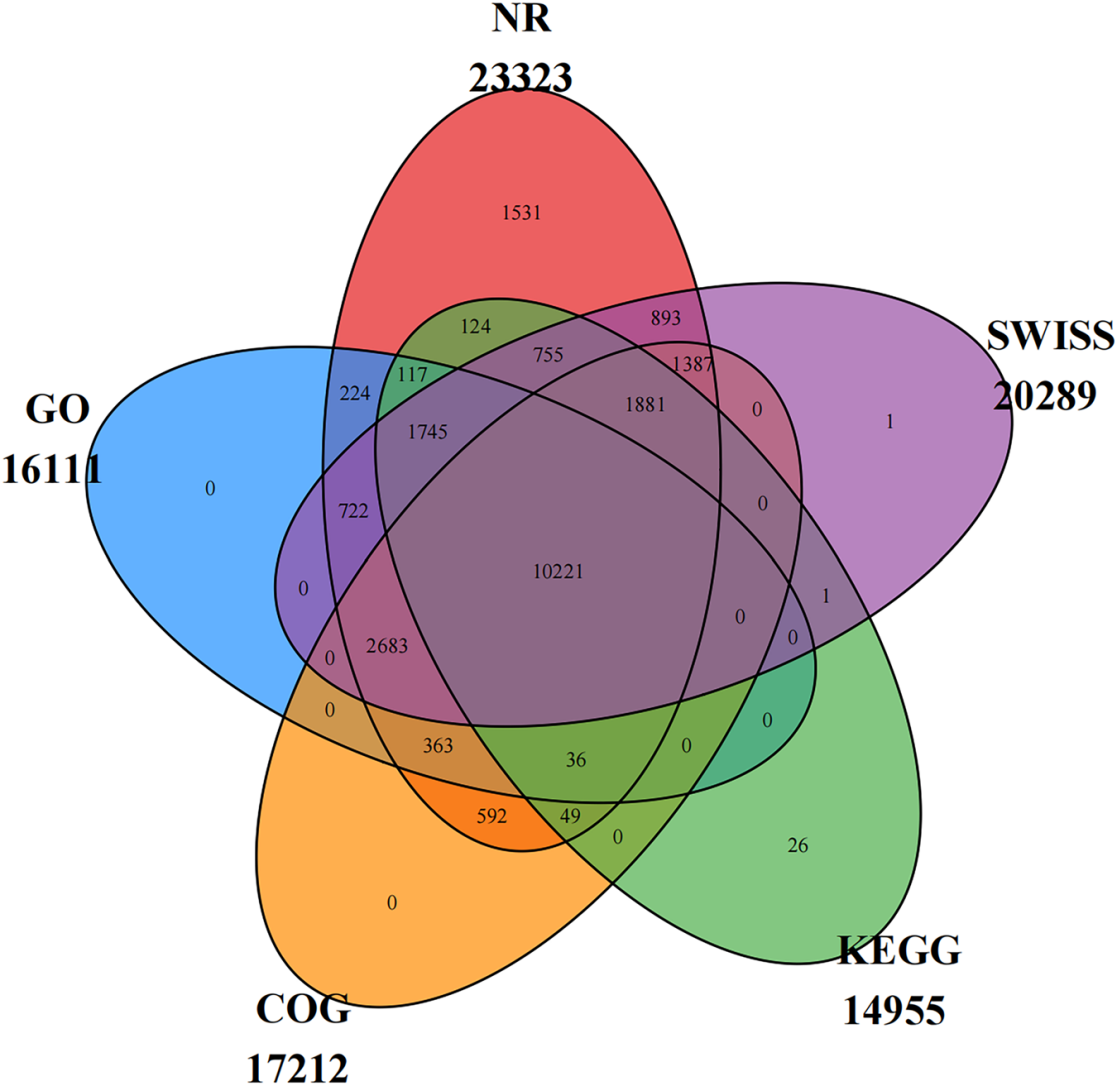
Venn diagram illustrating the number of functionally annotated genes across multiple public databases.

The protein sequences derived from the predicted genes were functionally annotated by querying the Nr (https://ftp.ncbi.nlm.nih.gov/blast/db/FASTA/nr.gz), GO^51^, KEGG^52^, eggNOG, and Swiss-Prot^53^ databases using BLASTp (BLAST + 2.7.1)^54^. A stringent E-value threshold of ≤1e-5 was applied to ensure the reliability of the matches. To maintain biological relevance, only the best match from potentially multiple results for each sequence comparison was retained as the database comparison information for the gene. Ultimately, we successfully annotated 23,351 unigenes, representing 79.93% of the total predicted genes, in at least one database (Table 9, Fig. 5).

**Table 9:** Statistical table of coding gene information.

DB_name	Total_unigenes	Annoted_unigenes	Percent (%)
NR	29,213	23,323	79.84
GO	29,213	16,111	55.15
COG	29,213	17,212	58.92
KEGG	29,213	14,955	51.19
SWISS	29,213	20,289	69.45
In_all_DB	29,213	10,221	34.99
AT_least_one_DB	29,213	23,351	79.93

## Data Records

The raw sequencing data used in this study included Illumina genomic reads (SRR32550678)^55^, PacBio long reads (SRR32550677)^56^, RNA-seq reads (SRR32550675)^57^, and Hi-C data (SRR32550676)^58^ all of which have been uploaded to the NCBI database. This whole genome shotgun project has been deposited at DDBJ/ENA/GenBank under the accession JBMUMZ000000000^59^. The assembly and annotation information has been uploaded to Figshare^60^.

## Technical Validation

### Data filtering and quality control

The preliminary quality evaluation of raw sequencing data was conducted using FastQC v0.11.8, as raw reads often contain low-quality segments. To enhance the accuracy of downstream assembly, Trimmomatic v0.39 was employed to trim the raw data. This process involved several steps: removing splice sequences from the reads, discarding bases with non-AGCT characters at the 5′ end prior to trimming, and trimming reads with low sequencing quality (defined as a quality score below Q20). Additionally, reads with more than 10% ambiguous bases (N) were eliminated, and fragments shorter than 75 base pairs were discarded after adapter removal and quality trimming. The resulting high-quality reads were saved in FASTQ format for subsequent analysis.

### Assembly validation

To ensure the precision and integrity of the assembled genome for subsequent functional annotation and cross-species genomic comparisons, a comprehensive quality assessment was conducted following the assembly process. The assembly quality was validated through a comprehensive approach comprising four methods: K-mer analysis, GC depth analysis, BUSCO assessment, and Merqury analysis. The application of 21-mer frequency analysis enabled the simultaneous determination of genomic parameters, including size, heterozygosity, and duplication rates, as well as the quality assessment of sequencing data. The distribution of GC content and sequencing coverage for the assembled sequences was examined through the GC depth distribution map. The BUSCO assessment was performed to evaluate the completeness and accuracy of the genome assembly by utilizing a set of highly conserved single-copy orthologous genes as references. Merqury is a genome assembly quality assessment tool that utilizes k-mer analysis to evaluate the accuracy (QV) and completeness of an assembly without depending on a reference genome.

## Data Availability

Unless otherwise specified, all software and tools used in this study were executed with their default parameters. No custom code or scripts were developed or applied during the course of this research.
